# Assessment of the abuse liability of three menthol Vuse Solo electronic cigarettes relative to combustible cigarettes and nicotine gum

**DOI:** 10.1007/s00213-018-4904-x

**Published:** 2018-05-03

**Authors:** Mitchell F. Stiles, Leanne R. Campbell, Tao Jin, Donald W. Graff, Reginald V. Fant, Jack E. Henningfield

**Affiliations:** 1 RAI Services Company, 401 N. Main Street, Winston-Salem, NC 27101 USA; 2 Celerion, Lincoln, NE USA; 3 PinneyAssociates, Inc., Bethesda, MD USA

**Keywords:** Abuse liability, Electronic cigarettes, Menthol, Nicotine pharmacokinetics, Subjective measures

## Abstract

**Rationale:**

We previously reported that following a short-term product use period, use of non-menthol Vuse Solo electronic cigarettes (ECs) resulted in product effect-related subjective responses and nicotine uptake between those of combustible cigarettes (high-abuse liability comparator) and nicotine gum (low-abuse liability comparator); the results were generally closer to those of nicotine gum.

**Objective:**

Using a similar design to the previous study, we evaluated the abuse liability of three menthol-flavored Vuse Solo ECs with the same nicotine contents (14, 29, and 36 mg) in a group of EC-naïve, menthol cigarette smokers, relative to comparator products.

**Methods:**

Six-hour nicotine uptake and ratings of subjective effects were used to determine abuse liability and pharmacokinetics.

**Results:**

Use of menthol Vuse Solo resulted in significantly lower responses to subjective measurements (product liking, intent to use product again, and liking of positive product effects), higher urge to smoke responses, and a lower peak (*C*_max_) and overall extent (AUC_0–360_) of nicotine uptake compared to smoking the usual brand menthol cigarette. When compared with use of nicotine gum, subjective responses to use of menthol Vuse ECs were in the same direction as those resulting from smoking cigarettes but were more similar to nicotine gum use in magnitude than they were to cigarettes.

**Conclusion:**

These findings are concordant with our previous results and provide evidence that menthol Vuse Solo ECs have abuse liability that is lower than menthol cigarettes and potentially greater than that of nicotine gum.

**Trial registration:**

ClinicalTrials.gov identifier: NCT02664012

**Electronic supplementary material:**

The online version of this article (10.1007/s00213-018-4904-x) contains supplementary material, which is available to authorized users.

## Introduction

The Family Smoking Prevention and Tobacco Control Act of 2009 requires the FDA to evaluate the impact that new tobacco products will have on public health of the US population (US DHHS 2010; US DHHS 2012; The Family Smoking Prevention and Tobacco Control Act 2009). The 2014 Surgeon General’s report and various tobacco control experts have concluded that alternative nicotine delivery products may be useful and appropriate to benefit public health (Abrams 2014; Hatsukami 2013; Shihadeh and Eissenberg 2015; Warner et al. 1997; Zeller 2013; Niaura 2016). Abuse liability assessments provide the FDA with one type of information regarding the capacity a new product has to impact the public health (US DHHS 2011; US DHHS 2012, 2016).

Abuse liability has been described as “the likelihood that individuals will engage in persistent and problematic use” of a drug and “the likelihood that individuals will experience undesirable consequences as a result of its use” (Carter et al. 2009). Abuse liability assessment can also be useful in assessing the potential of a product to contribute to the 2014 Surgeon General’s report goal of use in place of combustible cigarettes by providing sufficient nicotine in a sufficiently appealing manner to compete with cigarettes (Henningfield 2015). Combustible cigarettes have demonstrated a high degree of abuse liability and health risk to the consumer (US DHHS 2010, 2014). Electronic cigarettes (ECs) and their aerosol emissions have been characterized as having much lower levels of most of the toxicants commonly found in cigarette smoke (The National Academies of Sciences, Engineering, and Medicine 2018). Nicotine-containing vapor products need to deliver nicotine and provide a sufficient degree of acceptability and attraction, and therefore some abuse liability, to serve as viable and compelling alternatives for smokers (Abrams 2014; Abrams et al. 2017; Hatsukami 2013; Niaura 2016; Zeller 2013; Gottlieb and Zeller 2017). Stated somewhat differently, “If a particular product is far from cigarettes and close to NR [nicotine replacement] on the continuum of harm and at the same time closer to cigarettes than NR on the continuum of dependence, this product may have considerable success in reducing the public health costs associated with cigarette use” (Fagerström and Eissenberg 2012).

The Family Smoking Prevention and Tobacco Control Act of 2009 banned all characterizing flavors in combustible cigarettes with the exception of tobacco and menthol, although characterizing flavors in ECs are still allowed. Menthol is used in foods, drugs, and tobacco products to provide flavor, odor, and cooling effects (Henningfield et al. 2011; Lawrence et al. 2011; Wickham 2015). It is the most widely branded and popular characterizing flavor in cigarettes, with menthol cigarettes representing approximately a 26% share of market (2015) for cigarettes sold in the USA (Federal Trade Commission 2017). Menthol has been pointed to as influencing cigarette smoking behavior and the abuse liability of cigarettes (Lawrence et al. 2011; Benowitz et al. 2011; Ahijevych and Garrett 2004; Henningfield et al. 2011). We previously reported that three tobacco-flavored Vuse Solo ECs showed significantly lower abuse liability than combustible cigarettes, and a somewhat higher abuse liability than nicotine gum (Stiles et al. 2017). Building upon these findings among smokers of non-menthol, usual brand (UB) cigarettes, we performed the current study using a similar design to examine the same elements of abuse liability for three menthol-flavored Vuse Solo ECs (with smokers of menthol UB cigarettes) containing the same amounts of nicotine as those in the previous study. This provided an assessment of the abuse liability of commercially available menthol Vuse Solo ECs relative to the same two comparator products.

## Materials and methods

This was a randomized, open-label, cross-over study (ClinicalTrials.gov identifier: NCT02664012) completed at a single research center (Celerion, Lincoln, NE). The study was reviewed and approved by Chesapeake Institutional Review Board (Columbia, MD) and was conducted in accordance with the ethical standards in the Declaration of Helsinki and applicable sections of the US Code of Federal Regulations and ICH E6 Good Clinical Practices. With few exceptions, the design of the current study was identical to a previous study in which we evaluated non-menthol versions of similar Vuse Solo ECs (Stiles et al. 2017). A brief summary of the study methods is provided herein.

### Subjects

Potential subjects were recruited using standard advertising methods (print, radio, television, websites) and from an existing database of individuals who had previously participated, or who previously expressed interest in participating, in a clinical study. Study recruits were excluded from having participated in another clinical study within (≤) 30 days prior to screening. As in the former study, informed consent was obtained from all potential subjects prior to initiation of any study events. Eligibility criteria were assessed during a screening process to ensure that subjects were in generally good health, satisfied all requirements for inclusion, and met none of the criteria for exclusion. Subjects were 21 to 60 years of age and an attempt was made to enroll an approximate balance of males and females. Subjects self-reported smoking 10 or more menthol king size (83–85 mm) or 100-mm combustible, filtered cigarettes per day for at least 6 months prior to enrollment. They reported typically smoking their first cigarette of the day within 30 min of waking. Subjects were excluded if they reported current or recent regular use (i.e., any use within [≤] 30 days prior to screening) of ECs prior to entering the study.

### Investigational products

Three commercially available menthol Vuse Solo ECs (containing either 14, 29, or 36 mg of nicotine) were evaluated along with the subjects’ UB cigarette and Nicorette® White Ice Mint 4-mg nicotine polacrilex gum (GlaxoSmithKline Consumer Healthcare, L.P.) as the high- and low-abuse liability comparators, respectively. Nicotine yields in the product aerosols (mean ± standard deviation [SD]) for the three menthol Vuse Solo ECs (14 mg, 29 mg, 36 mg) were 1.02 ± 0.06 mg, 1.88 ± 0.15 mg, and 2.64 ± 0.28 mg, respectively. The analytical puffing regimen consisted of 20 puffs of a 55-ml volume, square-wave machine puff of 3-s duration, taken once every 30 s. The three ECs and nicotine gum were provided at no cost to subjects. Subjects provided their own UB cigarettes throughout the study. Menthol Vuse Solo ECs are composed of a battery, heating element, microchips, sensor, and a cartridge containing propylene glycol, glycerin, nicotine, flavorings (including menthol), and water. The three ECs were presented without commercial packaging and were therefore visually indistinguishable by subjects (i.e., differences in nicotine concentrations were not apparent from the products/cartridges).

### Study design

Eligible subjects who were enrolled into the study were randomized to one of 10 investigational product sequences based on a Williams Design. A 7-day ambulatory (“home use”) trial of each product preceded each of five, consecutive weekly test visits to allow subjects to become accustomed to using the assigned non-UB products. For at-home use, subjects were instructed to use the assigned product at least once on 6 of the 7 days preceding a test visit, with additional use permitted as desired. One “use” of Vuse Solo or nicotine gum was defined as approximately 10 to 30 min of ad libitum use, respectively, to approximate use in test visits. Subjects were not to use ECs or nicotine gum on Day 7 (the day prior to a test visit) to avoid potential impacts on subjective measures that might arise from residual nicotine absorption via the slower buccal route.

Smoking of UB cigarettes during each day of the at-home trial was allowed regardless of investigational product assignment, and all product use was tracked daily using an electronic diary. Subjects were instructed to abstain from all tobacco and nicotine products for at least 12 h prior to each test visit to minimize the impact that residual nicotine might have on baseline subjective and physiological measurements.

Subjects reported to the clinic on the morning of each test visit and were initially assessed for continued eligibility. Subjects with an expired carbon monoxide value > 12 ppm were not eligible to participate in the clinical procedures on that day but were allowed to reschedule one test visit for this reason. In-clinic use of one of the investigational products each week consisted of up to 10 min of ad libitum use of one of the menthol Vuse Solo ECs or smoking one cigarette, or up to 30 min of ad libitum use of nicotine gum according to the package instructions (i.e., “park and chew” method). In-clinic use of each of the three types of products occurred in separate sections of the clinic to minimize any potential effects of secondhand smoke or other sensory cues on subjective assessments. Subjective effects questionnaires, serial blood samples, and physiological measurements were collected at the specified time points relative to the start of product use as shown in Supplemental Table 1. Individual Vuse Solo cartridge weights, before (initial weight) and after (final weight) in-clinic use, were recorded to assess the amount of product use.

### Outcomes

Five subjective effects questionnaires were administered during test visits using an electronic tablet (CRFHealth, Hammersmith, UK). Product Liking, Urge to Smoke, and Urge for Product questionnaires were administered as 11-point numeric rating scales. The Product Effects questionnaires (Liking of Positive Effects and Disliking of Negative Effects) were administered as 10-point scales, and the Intent to Use Product Again questionnaire was administered as a 7-point vertical numeric scale. As in the previous study, Product Liking, Intent to Use Product Again, and Product Effects questionnaires were presented at 15, 30, 45, 60, 120, 180, 240, 300, and 360 min following the start of product use. The Urge to Smoke and Urge for Product questionnaires were administered at those and the additional time points of 5, 90, and 150 min. For the calculation of area under the effect curve (AUEC), a value of zero was assigned to any time points for which subjects responded as not feeling product effects (positive or negative) on the initial Yes/No question. A more detailed description of the questionnaires is included in the original study publication (Stiles et al. 2017). Urge for Product was not administered during UB cigarette use, so the data collected for Urge to Smoke from the cigarette condition was compared to the Urge for Product data from the EC and nicotine gum conditions.

Sample analysis for determination of serum nicotine and baseline cotinine concentrations was conducted by Celerion (Lincoln, NE) using a validated LC-MS/MS method. Pulse rate, systolic and diastolic blood pressure, and expired carbon monoxide were assessed as physiological measures related to product use. Baseline cotinine concentrations were measured at each test visit to assess whether subjects substantially changed their nicotine uptake during the study. All of these endpoints were measured as described in the earlier paper. As in the previous study, 18 blood samples were collected for measurement of nicotine concentrations, at the following times relative to the start of product use: − 5, − 0.5, 5, 7.5, 10, 15, 20, 30, 45, 60, 75, 90, 120, 150, 180, 240, 300, and 360 min. Cotinine concentrations were measured from the primary baseline sample (− 0.5 min) only, when available. Physical and oral examinations, clinical laboratory tests, vital sign measurements, electrocardiograms, and adverse events were used to assess safety and tolerability.

Serum nicotine concentrations below the limit of quantitation (0.20 ng/mL) were imputed to one-half the lower limit of quantitation for analysis. Additionally, observed and imputed nicotine concentrations were baseline-adjusted for the concentration of nicotine in the blood at the start of product use (Shiffman et al. 2009; Benowitz et al. 2006) to assess AUC (area under the curve), *C*_max_ (baseline-adjusted maximum plasma concentration), and *T*_max_ (time to baseline-adjusted maximum plasma concentration) uptake parameters.

### Statistical analyses

The target number of subjects needed to complete the study was based upon the findings of the previous study evaluating the non-menthol investigational products (Stiles et al. 2017). Fifty subjects were required in order to have 80% power to detect an effect size of 0.8 for the subjective measurements (which is equivalent to a mean difference of 0.8 and a standard deviation of 1.0) and at least a 20% absolute difference for the pharmacokinetic (PK) endpoints between each menthol Vuse Solo product and the high- and low-abuse liability comparator products. The comparisons of interest were results for each of the Vuse Solo ECs relative to the respective comparator products; the three ECs (14, 29, 36 mg) were not compared to each other. Statistical significance is indicated for *p* values below 0.05.

Data management and statistical analyses were performed by Celerion (Lincoln, NE). Phoenix® WinNonlin® Version 6.3 (Pharsight, Princeton, NJ) was used to calculate non-compartmental PK and subjective measure response parameters. Statistical summarizations and comparisons were calculated using SAS® Version 9.3 (SAS, Cary, NC).

A mixed-effect model analysis of variance (ANOVA) was used to compare maximum effect (*E*_max_) values of product liking, intent to use product again, (liking of) positive effects and (disliking of) negative effects, the product liking area under the effect curve (AUEC_15–360_), the plasma nicotine PK parameters (AUC_0–15_, AUC_0–360_, *C*_max_, and *T*_max_), baseline cotinine, and the maximum absolute change in physiological measures (pulse rate and systolic and diastolic blood pressures). Except for *T*_max_, the PK parameters were analyzed on the natural log scale. Sequence, period, and product were included as fixed effects, and subject-nested-within-sequence was included as a random effect in each model. Results are presented as geometric least-squares means for all PK parameters except for *T*_max_, which are presented as least-squares means.

A mixed-effect model analysis of covariance (ANCOVA) was used to compare urge to smoke AUEC_0–15_, AUEC_0–360_, and *E*_min_, and urge for product AUEC_0–360_ and *E*_max_. Sequence, period, product, and the baseline score were included as fixed effects and subject-nested-within-sequence was included as a random effect in each model. These results are presented as least-squares means. Lastly, a paired *t* test was used to detect changes in expired carbon monoxide between the baseline and post-product-use measurements.

## Results

### Subjects

One hundred eleven subjects were screened for study participation, 71 subjects were randomized to investigational product use sequences, and 55 subjects completed all five test visits. Eleven subjects withdrew consent from the study, two subjects were lost to follow-up, two subjects were discontinued by the principal investigator due to protocol deviations, and one subject was discontinued due to a pregnancy. Demographic data are summarized in Supplemental Table 2. Almost two thirds of the subjects were male and one third of subjects were African American. Various menthol Marlboro (*n* = 26, 37%), Newport (*n* = 21, 30%), and Pall Mall (*n* = 8, 11%) brand styles were the most commonly reported UB cigarettes smoked by randomized subjects. No subject reported current or recent regular use of ECs prior to entering the study. Results include all available data for all subjects with evaluable PD or PK profiles.

### Subjective measures

Product Liking *E*_max_ for the menthol Vuse Solo products ranged from 4.51 to 5.08 and was significantly lower compared to UB smoking (*E*_max_ 9.29, *p* < 0.001 for all) and somewhat higher than with nicotine gum (*E*_max_ 3.25, *p* < 0.005 for all). Intent to Use Again *E*_max_ followed a similar pattern, with use of the Vuse Solo ECs ranging from 4.25 to 4.49, which was significantly lower compared to UB smoking (6.93, *p* < 0.0001 for all) and higher than nicotine gum (3.32, *p* < 0.005 for all). Urge for Product *E*_max_ ranged from 4.52 to 4.82 with use of Vuse Solo ECs, which was significantly higher compared to using nicotine gum (3.62, *p* < 0.05 for all). Among subjects who reported liking of positive effects, *E*_max_ was also significantly lower for the three Vuse Solo ECs, ranging from 6.44 to 6.74, compared to UB smoking (8.63, *p* ≤ 0.0005), but there were no differences compared to the nicotine gum (6.02). Among subjects who reported negative effects, there were no significant differences detected for disliking of negative effects with Vuse Solo ECs (5.16 to 6.16) and smoking (6.06) or compared to use of nicotine gum (6.24). Results for AUEC_15–360_ for all subjective measures are shown in Table 1 and were largely in agreement with the *E*_max_ comparisons.

**Table 1:** Statistical comparisons of subjective measure parameters

	LS means (95% confidence intervals)				
Parameter (95% confidence interval)	Menthol Vuse Solo 14 mg	Menthol Vuse Solo 29 mg	Menthol Vuse Solo 36 mg	Usual brand cigarette	Nicotine gum
Product Liking (AUEC_15–360_)	1521.63^†§^ (1314.14, 1729.12)	1426.20^†§^ (1204.32,1648.08)	1256.89^†§^ (1035.52, 1478.27)	3148.10 (2933.18, 3363.02)	907.29 (692.69, 1121.89)
*E*_max_	5.08^†§^ (4.46, 5.70)	4.51^†^ (3.86, 5.16)	4.53^†^ (3.86, 5.19)	9.29 (8.65, 9.93)	3.25 (2.61, 3.89)
Intent to Use Again (AUEC_15–360_)	1489.01^†§^ (1346.90, 1631.12)	1534.54^†§^ (1383.20, 1685.87)	1412.88^†§^ (1261.88, 1563.89)	2403.50 (2256.57, 2550.43)	1143.37 (996.69, 1290.05)
*E*_max_	4.40^†§^ (3.99, 4.80)	4.49^†§^ (4.06, 4.91)	4.25^†^ (3.82, 4.68)	6.93 (6.52, 7.35)	3.32 (3.82, 4.68)
Liking of Positive Effects (AUEC_15–360_)	766.72^†^ (475.9, 1057.54)	1003.47^†^ (709.08, 1297.87)	704.70^†^ (400.05, 1009.36)	1388.31 (1102.92, 1673.70)	842.96 (542.72, 1143.21)
*E*_max_	6.45^†^ (5.79, 7.11)	6.44^†^ (5.76, 7.12)	6.74^†^ (6.01, 7.47)	8.63 (8.00, 9.27)	6.02 (5.32, 6.72)
Disliking of Negative Effects (AUEC_15–360_)	596.25 (297.04, 895.46)	822.23 (512.69, 1131.77)	491.65 (207.8, 775.51)	787.93 (462.74, 1113.12)	771.89 (498.84, 1044.94)
*E*_max_	5.16 (4.15, 6.17)	6.16 (5.10, 7.21)	5.17 (4.23, 6.11)	6.06 (4.94, 7.17)	6.24 (5.34, 7.13)

^†^Significantly different from usual brand cigarette; *p* < 0.05

^§^Significantly different from nicotine gum; *p* < 0.05

### Urge to smoke

**Fig. 1:**
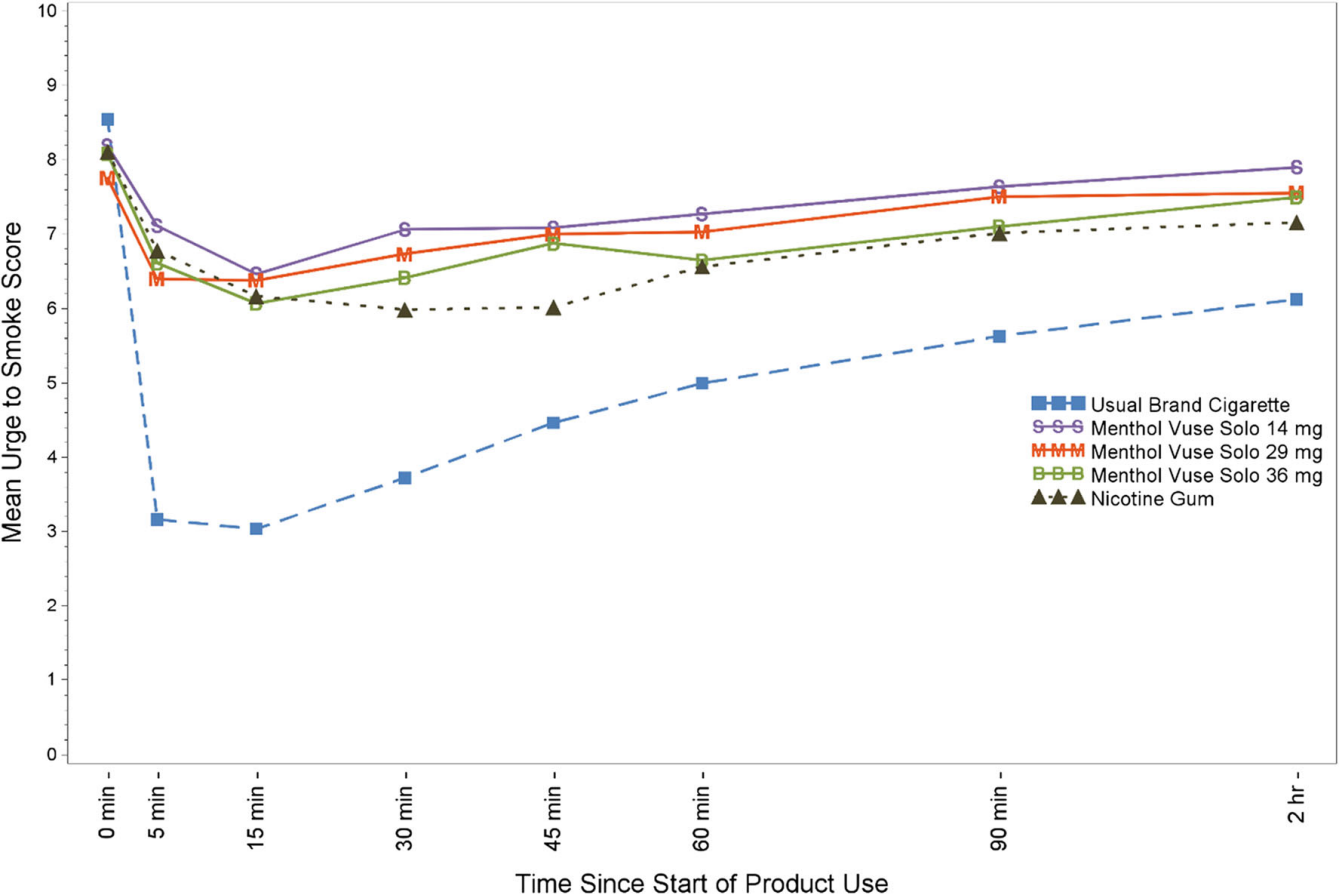
Mean ratings for the urge to smoke question “How strong is your current urge to smoke your usual brand cigarette?”

As illustrated in Fig. 1 (first 2 h of test visit), urge to smoke decreased more rapidly and to a greater extent following smoking UB compared to use of menthol Vuse Solo ECs and nicotine gum, but the curves began to converge by the end of the 6-h session (data not shown in Fig. 1). Urge to Smoke scores were higher through the first 15 min with all Vuse Solo ECs (AUEC_0–15_ ranging from 103.70 to 106.08) compared to smoking (AUEC_0–15_ = 59.58, *p* < 0.0001), whereas the scores with all of the Vuse Solo ECs were not significantly different from nicotine gum (AUEC_0–15_ = 104.25). Urge to Smoke scores over the 6-h session (AUEC_0–360_) were also significantly higher with Vuse Solo ECs (AUEC_0–360_ = 2802.17 to 2873.61) compared to smoking (AUEC_0–360_ = 2302.64, *p* < 0.0001). There were no statistically significant differences in Urge to Smoke scores when nicotine gum (AUEC_0–360_ = 2688.18) was compared to the 14-mg Vuse Solo (AUEC_0–360_ = 2852.04) and 36-mg Vuse Solo (AUEC_0–360_ = 2802.17); however, use of the 29-mg Vuse Solo did result in a significantly higher score (AUEC_0–360_ = 2873.61; *p* < 0.05).

The time to reach the minimum Urge to Smoke (*T*_min_) was not significantly different between the three Vuse Solo ECs (*T*_min_ ranging from 20.36 to 24.78 min) and either the UB cigarettes (*T*_min_ = 16.17 min) or the nicotine gum (*T*_min_ = 24.52 min).

### Nicotine pharmacokinetics

**Fig. 2:**
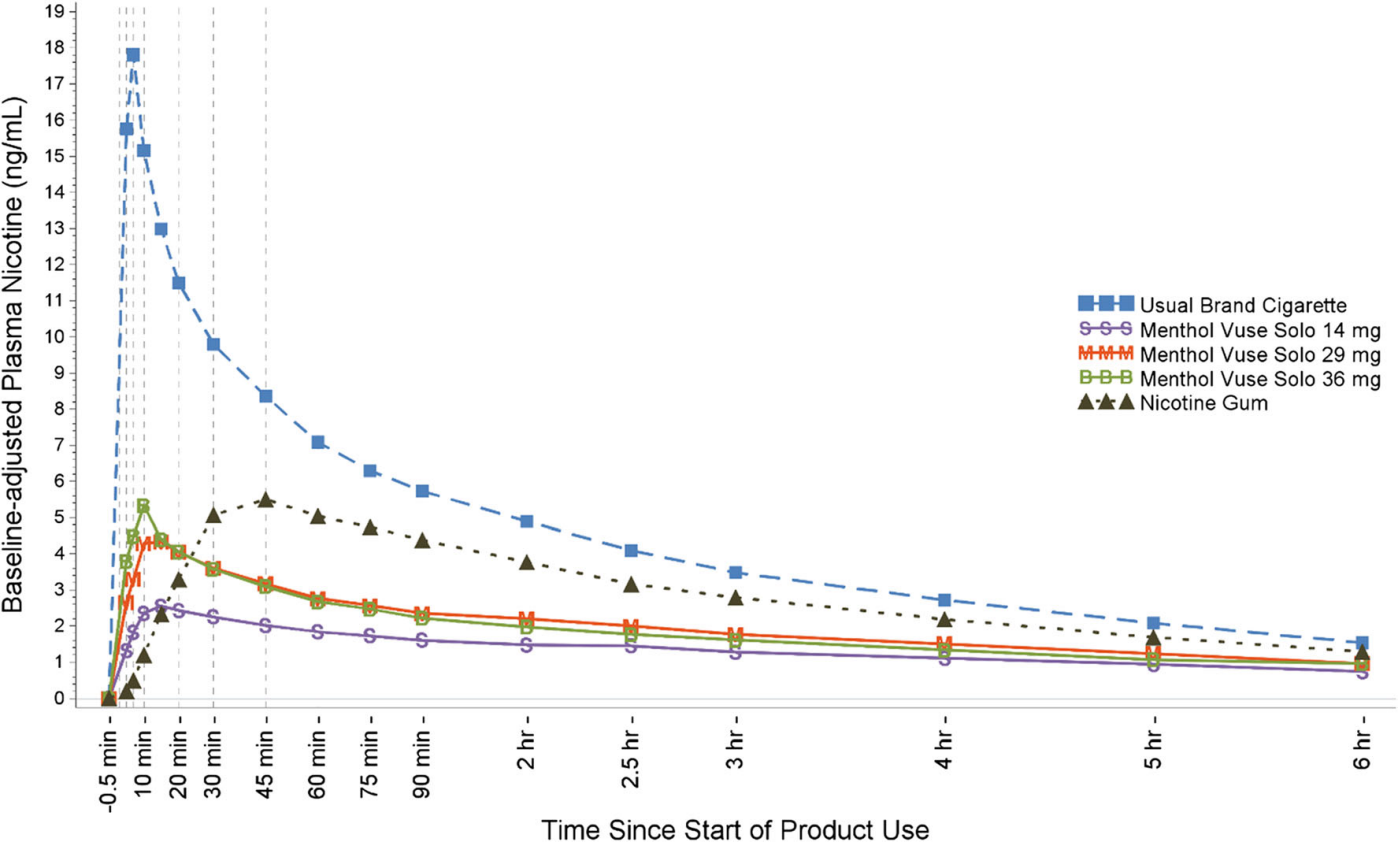
Mean plasma nicotine concentration profiles

As illustrated in Fig. 2, nicotine concentrations increased rapidly within 15 min of smoking and with use of each of the menthol Vuse Solo ECs and more gradually with use of the nicotine gum (which peaked around 45 min). By 6 h, blood levels had declined to near convergence at about 1 ng/ml. Baseline-adjusted nicotine PK parameters are summarized in Table 2. Nicotine uptake was significantly lower with use of the three Vuse Solo ECs (AUC_0–15_ = 17.14 to 33.14) compared to smoking (176.30), and significantly higher compared to the nicotine gum (8.44), during the first 15 min following the start of product use (*p* < 0.0001 for each comparison to Vuse Solo). Overall nicotine uptake, based on AUC_0–360_, was significantly lower with the three Vuse Solo ECs (412.34 to 545.14) compared to both smoking (1556.44) and nicotine gum (844.01), with *p* < 0.0001 for each comparison to Vuse Solo. Similarly, *C*_max_ was significantly lower with use of each Vuse Solo EC compared to smoking (18.04 ng/ml, *p* < 0.0001 for each comparison). Compared to nicotine gum (4.80 ng/ml), *C*_max_ was significantly lower (*p* < 0.005) for Vuse Solo 14 mg (2.45 ng/ml), but not for Vuse Solo 29 mg (3.40 ng/ml) or Vuse Solo 36 mg (3.94 ng/ml). In addition, *T*_max_ was significantly shorter with the UB cigarette (7.43 min) compared to the Vuse Solo ECs (10.13 to 19.89 min), *p* < 0.0005 for each comparison, and was significantly shorter for the Vuse Solo ECs compared to the nicotine gum (45.04 min), *p* < 0.01 for each comparison.

**Table 2:** Statistical comparisons of baseline-adjusted plasma nicotine uptake parameters

	Geometric LS means^1^ (95% confidence intervals)				
Parameter	Menthol Vuse Solo 14 mg	Menthol Vuse Solo 29 mg	Menthol Vuse Solo 36 mg	Usual brand cigarette	Nicotine gum
*C*_max_ (ng/ml)	2.45^†§^ (2.08, 2.90)	3.40^†^ (2.87, 4.02)	3.94^†^ (3.32, 4.67)	18.04 (15.20, 21.41)	4.80 (4.06, 5.69)
AUC_nic0–15_ (ng x min/ml)	17.14^†§^ (14.11, 20.82)	26.26^†§^ (21.58, 31.95)	33.14^†§^ (27.12, 40.49)	176.30 (144.45, 215.19)	8.44 (6.93, 10.27)
AUC_nic0–360_ (ng x min/ml)	412.34^†§^ (358.31, 474.52)	545.14^†§^ (473.14, 628.1)	516.15^†§^ (447.11, 595.86)	1556.44 (1347.9, 1797.23)	844.01 (732.23, 972.92)
*T*_max_ (minutes)	19.89^†§^ (15.35, 29.93)	15.10^†§^ (14.89, 19.97)	10.13^†§^ (9.97, 14.92)	7.43 (6.95, 7.52)	45.04 (44.96, 46.54)

^1^*T*_max_ presented as median

^†^Significantly different from usual brand cigarette; *p* < 0.05

^§^Significantly different from nicotine gum; *p* < 0.05

### Baseline plasma cotinine concentrations

No differences were noted in baseline plasma cotinine concentrations (LS means range = 209.19 to 218.65 ng/mL) across study visits, indicating that overall nicotine uptake did not change throughout the study with the investigational use of Vuse Solo ECs and nicotine gum.

### Product use

Use of comparator products during the test visits included ad libitum smoking of a single UB cigarette within a 10-min period and use of a single piece of nicotine gum for up to 30 min. Based on the mean pre- to post-use differences in e-liquid cartridge weights, from ad libitum use of Vuse Solo ECs for up to 10 min, subjects tended to use slightly more of Vuse Solo 14 mg (0.021 g), followed by Vuse Solo 29 mg (0.019 g) and Vuse Solo 36 mg (0.013 g). Summary statistics for at-home use (with subject-specific averages as input values) of all products are presented in Supplemental Table 3. Subjects used UB cigarette most frequently on a daily basis. Distributions of daily use for the Vuse Solo ECs were fairly similar from the minimum through the 3rd quartile, and so were the mean values.

### Physiological effects

Pulse rates and systolic and diastolic blood pressures at baseline were similar with use of each of the investigational products, ranging from a mean of 63.8 to 65.3 bpm, 115.1 to 117.6 mmHg, and 67.9 to 71.2 mmHg, respectively. As shown in Supplemental Table 4, use of the Vuse Solo 14 mg and Vuse Solo 36 mg resulted in smaller absolute changes in pulse rate compared to smoking UB cigarettes (*p* < 0.05), whereas there were no significant differences in absolute changes in pulse rate after use of Vuse Solo ECs compared to use of the nicotine gum. There were also no statistically significant differences in the absolute changes in blood pressure with use of the menthol Vuse Solo ECs vs. the comparator products.

### Expired carbon monoxide

Baseline expired carbon monoxide mean values were comparable prior to use of each investigational product, ranging from 6.87 to 7.53 ppm. As expected, the difference from baseline value was relatively unchanged following use of the three Vuse Solo ECs and nicotine gum (differences ranging from − 0.25 to + 0.35 ppm), but the increase from baseline was significant following use of the UB cigarette (5.08 ppm, *p* < 0.0001). With regard to the baseline measurement, although subjects were instructed to refrain from use of tobacco/nicotine products for at least 12 h prior to test visits, their use of ECs or nicotine gum would not be detected by this assessment.

### Safety

The investigational products were well-tolerated under the conditions of use during the study. Fifty-nine adverse events were reported by 28 of the 71 subjects. All adverse events were mild in severity; 11 were considered to be related to study product use and four were considered to be possibly related. Headache was the most common adverse event reported during this study, with 10 episodes reported by 10 subjects. All other adverse events were reported by five or fewer subjects each. The number of adverse events reported was comparable across investigational products, ranging from 9 to 13 with use of the three Vuse Solo ECs, 14 with the UB cigarette, and 11 with the nicotine gum.

## Discussion

Electronic cigarette use is on the rise as many smokers look for potentially lower-health-risk products in an effort to displace cigarette use. In order to successfully replace more toxic combustible products, potentially lower-risk products must possess characteristics that are desirable to smokers. As stated by Gottlieb and Zeller (2017, p. 1), “…potentially less harmful tobacco products could reduce risk while delivering satisfying levels of nicotine for adults who still need or want it.” “Satisfying levels of nicotine” implies some level of abuse liability, but the level that is acceptable or desirable has not been defined by the FDA. Nonetheless, the FDA recommends that abuse liability should be assessed to support premarket tobacco applications, and will consider these data among other information in its evaluation (US DHHS 2016).

To our knowledge, there have been no well-controlled studies to evaluate the abuse liability of menthol ECs to date. The methods used to evaluate the abuse liability of menthol Vuse Solo ECs in the current study were adapted from the pharmaceutical model and are generally consistent with other researchers investigating ECs (Carter et al. 2009; FDA 2010, 2017; McColl and Sellers 2006; Vansickel et al. 2012). The study methods included a number of subjective measures as well as measurements of nicotine uptake that could indicate the potential for these products to be acceptable commercial alternatives to cigarette smoking. As in our non-menthol abuse liability study, use of UB cigarettes seemed a more conservative approach for the study design. Inclusion of a common (non-UB) combustible comparator might have biased positive subjective measures in a manner suggesting weaker positive effects, if subjects liked it less or very little relative to UB. With the exception of the e-liquid flavors in the EC investigational products, the designs and conclusions of our two studies were similar overall. For smokers of mentholated cigarettes in this study, the abuse liability of menthol Vuse Solo ECs falls between that of combustible cigarettes and nicotine gum.

Menthol ECs represent a major category of ECs for which a formal assessment of abuse liability is lacking. Comparisons across our two abuse liability studies were not a planned objective and therefore must be made with caution. Formal statistical comparisons were not performed between the two studies; however, a few observations seem worthy of note and potentially, of additional research.

Smokers of menthol cigarettes scored all menthol Vuse Solo ECs significantly lower across the test visit for ratings of Product Liking, Intent to Use Product Again, and Liking of Positive Effects (AUEC_15–360_ in all cases) when compared to UB cigarette (Table 3). These scores were also significantly higher than those for nicotine gum for the first two of those subjective measures. Both sets of results are in alignment with those measured previously for non-menthol smokers. Menthol and non-menthol smokers, respectively, also showed similar Disliking of Negative Effects (AUEC_15–360_) scores, and there were no significant differences in scores for the Vuse Solo ECs when compared to those for cigarettes or gum. Urge to Smoke scores were higher with all menthol Vuse Solo ECs and nicotine gum compared to scores after smoking UB cigarettes. This pattern was true both in the first 15 min of the test visit and across the entire testing period and was similar to that seen in the last study.

**Table 3:** Subjective measure parameters from the current menthol and previous non-menthol studies

	Vuse Solo 14 mg		Vuse Solo 29 mg		Vuse Solo 36 mg		Usual brand cigarette		Nicotine gum	
Parameter^1^	Menthol	Non-menthol	Menthol	Non-menthol	Menthol	Non-menthol	Menthol	Non-menthol	Menthol	Non-menthol
Product Liking (AUEC_15–360_)	1521.63^*,†^	1396.68	1426.20^*,†^	1430.66	1256.89^*,†^	1190.01	3148.10	3116.52	907.29	799.38
*E*_max_	5.08^*,†^	4.36	4.51^*,†^	4.57	4.53^*,†^	4.13	9.29	9.06	3.25	3.21
Intent to Use Again (AUEC_15–360_)	1489.01^*,†^	1619.43	1534.54^*,†^	1635.82	1412.88^*,†^	1400.99	2403.50	2369.30	1143.37	1091.84
*E*_max_	4.40^*,†^	4.71	4.49^*,†^	4.75	4.25^*,†^	4.07	6.93	6.81	3.32	3.29
Liking of Positive Effects (AUEC_15–360_)	766.72^*^	727.42	1003.47^*^	800.57	704.70^*^	673.67	1388.31	889.74	842.96	444.17
*E*_max_	6.45^*^	6.71	6.44^*^	6.51	6.74^*^	5.99	8.63	8.31	6.02	5.47
Disliking of Negative Effects (AUEC_15–360_)	596.25	502.66	822.23	827.41	491.65	740.85	787.93	423.38	771.89	422.14
*E*_max_	5.16	6.03	6.16	6.41	5.17	6.67	6.06	5.80	6.24	6.28
Urge to Smoke (AUEC_0–15_)	106.08^*^	97.07	104.15^*^	94.52	103.70^*^	104.38	59.58	60.52	104.25	107.35
Urge to Smoke (AUEC_0–360_)	2852.04^*^	2781.63	2873.61^*^	2715.24	2802.17^*^	2823.38	2302.64	2290.86	2688.18	2773.64
*E*_min_	5.76^*^	5.17	5.91^*^	4.97	5.59^*^	5.92	1.94	2.12	5.38	5.39

^1^Parameters presented as LS means

^*^Statistically significantly different than usual brand cigarette, *p* < 0.05

^†^Statistically significantly different than nicotine gum, *p* < 0.05

With the exception of *C*_max_ for menthol Vuse Solo 36 mg (not statistically significantly different from gum), the nicotine uptake parameters *C*_max_ and AUC_nic0–360_ were significantly lower for the menthol Vuse Solo ECs compared to the cigarette condition and the nicotine gum condition (Supplemental Table 5). However, as also noted in the previous study, early nicotine uptake in the first 15 min (AUC_0–15_) following the start of product use was statistically highest with UB and was significantly higher with Vuse ECs than with nicotine gum. Based on work by Shiffman et al. (1996), more rapid early absorption of nicotine could be helpful in preventing a relapse to smoking. For *T*_max_, the Vuse ECs were higher than the cigarette condition and lower than the nicotine gum condition. Generally speaking, these results were also observed in the previous study of non-menthol smokers.

Results for nicotine uptake parameters for the UB cigarette and nicotine gum were generally consistent in both studies. Of note, all PK results with menthol Vuse Solo ECs in this study were directionally lower than those in the previous work, with differences ranging from approximately − 15% to approximately − 60%. The menthol study subjects included, respectively, a lower percentage of females than the prior study (38 vs. 42%), and a higher percentage of African Americans (31 vs. 0%). Menthol smokers were younger (mean age 34.3 vs. 39.7 years) and had higher body mass index (mean 29.0 vs. 27.1). They also smoked fewer cigarettes per day (mean 18.6 vs. 20.6) and had lower Fagerström Test for Cigarette Dependence scores (mean 5.4 vs. 5.8). If the differences in nicotine uptake were due to demographic variations between the two groups of subjects, we would expect the differences to also be present with the cigarette and gum conditions.

The primary intended audience for use of ECs is established adult smokers of combustible cigarettes, who are encouraged to migrate from use of combustibles to products that are generally recognized to potentially pose a lower risk of harm (Goniewicz et al. 2014; Royal College of Physicians 2016; Gottlieb and Zeller 2017). The authors acknowledge that established adult smokers are only one group of people who might experiment with and continue to use ECs. Distinct drivers may contribute to the use of ECs, such as desire for a product that helps to reduce or quit smoking, presents less risk/harm compared to smoking, provides a reduction in urge to smoke or withdrawal symptoms, and offers enjoyment factors (e.g., product design, taste) (Tucker et al. 2017; Baweja et al. 2016; Biener and Hargraves 2015; Harrell et al. 2015; Etter and Bullen 2011).

With many possible elements to consider across the abuse liability paradigm, a single study design, consistent with classical abuse liability methodology, can necessarily incorporate only limited objectives. The current study design was intended to be representative of the “real world” setting when a smoker first considers use of ECs, and assessed some of those products against two comparators that have long been commercially available. Established adult smokers have the greatest incentive to try existing or novel ECs. We acknowledge that testing other groups (e.g., experienced EC users, non-tobacco users, or former tobacco users) could have yielded different results from those presented here.

In summary, the results of the current study are in close agreement with our previous evaluation of non-menthol Vuse Solo ECs. Specifically, the results indicate that the abuse liability for the menthol Vuse Solo ECs tested in this study is markedly lower than that of combustible cigarettes, but somewhat higher than and closer to that of nicotine gum. From a public health perspective, the primary importance of menthol in electronic cigarettes and other electronic nicotine delivery systems may be in providing a commercially available alternate flavor category to offer an appealing option for smokers of menthol cigarettes (see general discussion in Abrams et al. 2017). In a diverse marketplace, Vuse Solo ECs are but one platform in terms of form and functionality. Continued research will provide a better understanding of the rapidly evolving vapor category’s utility to displace combustible cigarettes and potentially benefit public health.

## Electronic supplementary material

ESM 1 (DOCX 41 kb)
