# Supplementary material for: Assessment of the abuse liability of three menthol Vuse Solo electronic cigarettes relative to combustible cigarettes and nicotine gum
Source: Psychopharmacology (Berl). 2018 May 3;235(7):2077–86. doi: 10.1007/s00213-018-4904-x (PMC6015619; doi:10.1007/s00213-018-4904-x)
Supplement: Supplementary file 1 — (DOCX 41 kb) [file 213_2018_4904_MOESM1_ESM.docx]

**Supplementary Material File**

**Article Title:** Assessment of the abuse liability of three menthol Vuse Solo electronic cigarettes relative to combustible cigarettes and nicotine gum

**Journal Name:**  *Psychopharmacology*

**Authors and Affiliations:** Mitchell F. Stiles^1^, Leanne R. Campbell^1^, Tao Jin^1^, Donald W. Graff^2^, Reginald V. Fant^3^, Jack E. Henningfield^3^

^1^RAI Services Company, Winston-Salem, NC, USA

^2^Celerion, Lincoln, NE, USA

^3^Pinney Associates, Inc., Bethesda, MD, USA

**Corresponding Author:** Leanne R. Campbell; RAI Services Company; [campbel1@rjrt.com](mailto:campbel1@rjrt.com)

**Supplemental Table 1. Schedule of test visit events**

| Study Event | Time | BL | | 5 | | 7.5 | | 10 | | 15 | | 20 | | 30 | | 35 | | 45 | | 60 | | 75 | | 90 | | 120 | | 150 | | 180 | | 240 | | 300 | | 360 |
| --- | --- | --- | --- | --- | --- | --- | --- | --- | --- | --- | --- | --- | --- | --- | --- | --- | --- | --- | --- | --- | --- | --- | --- | --- | --- | --- | --- | --- | --- | --- | --- | --- | --- | --- | --- | --- |
| Product Liking | |  |  | |  | |  | | X | |  | | X | |  | | X | | X | |  | |  | | X | |  | | X | | X | | X | | X | |
| Intent to Use Again | |  |  | |  | |  | | X | |  | | X | |  | | X | | X | |  | |  | | X | |  | | X | | X | | X | | X | |
| Product Effects | |  |  | |  | |  | | X | |  | | X | |  | | X | | X | |  | |  | | X | |  | | X | | X | | X | | X | |
| Urge to Smoke | | X | X | |  | |  | | X | |  | | X | |  | | X | | X | |  | | X | | X | | X | | X | | X | | X | | X | |
| Urge for Product | | X | X | |  | |  | | X | |  | | X | |  | | X | | X | |  | | X | | X | | X | | X | | X | | X | | X | |
| Blood Sampling | | X | X | | X | | X | | X | | X | | X | |  | | X | | X | | X | | X | | X | | X | | X | | X | | X | | X | |
| Pulse Rate and Blood Pressure | | X |  | |  | |  | | X | |  | | X | |  | | X | | X | |  | |  | | X | |  | | X | | X | | X | | X | |
| Expired Carbon Monoxide | |  |  | |  | |  | |  | |  | |  | | X | |  | |  | |  | |  | |  | |  | |  | |  | |  | |  | |

BL=Baseline (baseline time points for blood sampling included -5 and -0.5 minutes relative to start of product use; baseline subjective measures and physiological measurements were also collected prior to blood sampling and product use).

Time (minutes) expressed relative to the start of product use.

Urge for Product was administered only during Menthol Vuse Solo EC and nicotine gum test visits.

**Supplemental Table 2. Demographic summary**

| **Sex, n (%)** |  |
| --- | --- |
| Female | 27 (38%) |
| Male | 44 (62%) |
| **Race, n (%)** |  |
| Asian | 2 (3%) |
| Black or African American | 22 (31%) |
| White | 44 (62%) |
| Other/mixed | 3 (4%) |
| **Ethnicity** |  |
| Hispanic or Latino | 3 (4%) |
| Not Hispanic or Latino | 68 (96%) |
| **Age, years** |  |
| Mean | 34.3 |
| SD | 10.12 |
| **Body Mass Index, kg/m²** |  |
| Mean | 29.0 |
| SD | 4.84 |
| **Cigarettes per Day** |  |
| Mean | 18.6 |
| SD | 14.38 |
| **Fagerström Test for Cigarette Dependence Score** | |
| Mean | 5.4 |
| SD | 1.42 |

**Supplemental Table 3: Summary of Daily At-Home Investigational Product Use**

| **Product** | **N** | **Mean ± SD** | **Minimum** | **1st Quartile** | **Median** | **3rd Quartile** | **Maximum** |
| --- | --- | --- | --- | --- | --- | --- | --- |
| **Usual Brand Cigarettes** | 71 | 14.65 ± 3.87 | 5.14 | 12.19 | 14.65 | 17.10 | 25.54 |
| **Menthol VUSE Solo 14mg** | 64 | 2.56 ± 3.17 | 0.80 | 1.00 | 1.43 | 2.29 | 15.67 |
| **Menthol VUSE Solo 29mg** | 63 | 2.30 ± 2.81 | 0.80 | 1.00 | 1.29 | 2.43 | 19.14 |
| **Menthol VUSE Solo 36mg** | 65 | 2.71 ± 3.89 | 0.60 | 0.86 | 1.14 | 2.60 | 23.40 |
| **Nicotine Gum** | 65 | 1.24 ± 1.19 | 0.71 | 0.86 | 0.86 | 1.29 | 10.00 |
| All available data from all subjects were included in the analysis.  Data from the Usual Brand Cigarettes included all available weeks. | | | | | | | |

Supplemental Table 4. Mean absolute change in pulse rate and blood pressure from baseline

|  |  | | | **Menthol Vuse Solo ECs vs  Usual Brand Cigarette** | | | | **Menthol Vuse Solo ECs vs  Nicotine Gum** | | | |
| --- | --- | --- | --- | --- | --- | --- | --- | --- | --- | --- | --- |
| **Parameter** | **LS Means of Maximum Absolute Change** | | | **Difference**  **(90% CI)** | | **p-Value** | | **Difference**  **(90% CI)** | | **p-Value** | |
| **Pulse Rate (bpm)** |  |  |  | |  | |  | |  | |  |
| Menthol Vuse Solo 14 mg | 15.12 | | | -3.11 | | 0.0080 | | 0.53 | | 0.6461 | |
|  |  |  |  | (-5.04 - -1.19) | |  | | (-1.38 - 2.45) | |  | |
| Menthol Vuse Solo 29 mg | 16.08 | | | -2.15 | | 0.0729 | | 1.50 | | 0.2101 | |
|  |  |  |  | (-4.13 - -0.18) | |  | | (-0.47 - 3.46) | |  | |
| Menthol Vuse Solo 36 mg | 15.29 | | | -2.94 | | 0.0159 | | 0.71 | | 0.5577 | |
|  |  |  |  | (-4.94 - -0.94) | |  | | (-1.28 - 2.69) | |  | |
| Usual Brand Cigarette | 18.23 | | |  | | | | | | | |
| Nicotine Gum | 14.58 | | |  | | | | | | | |
| **Systolic Blood Pressure (mmHg)** | | | | | | | | | | | |
| Menthol Vuse Solo 14 mg | 20.01 | | | 1.40 | | 0.3792 | | -1.16 | | 0.4669 | |
|  |  |  |  | (-1.23 - 4.03) | |  | | (-3.78 - 1.47) | |  | |
| Menthol Vuse Solo 29 mg | 20.19 | | | 1.58 | | 0.3358 | | -0.98 | | 0.5475 | |
|  |  |  |  | (-1.13 - 4.28) | |  | | (-3.67 - 1.71) | |  | |
| Menthol Vuse Solo 36 mg | 19.82 | | | 1.21 | | 0.4649 | | -1.35 | | 0.4138 | |
|  |  |  |  | (-1.52 - 3.95) | |  | | (-4.07 - 1.37) | |  | |
| Usual Brand Cigarette | 18.61 | | |  | | | | | | | |
| Nicotine Gum | 21.17 | | |  | | | | | | | |
| **Diastolic Blood Pressure (mmHg)** | | | | | | | | | | | |
| Menthol Vuse Solo 14 mg | 17.13 | | | 0.97 | | 0.4519 | | 1.34 | | 0.2956 | |
|  |  |  |  | (-1.15 - 3.09) | |  | | (-0.77 - 3.46) | |  | |
| Menthol Vuse Solo 29 mg | 17.45 | | | 1.29 | | 0.3305 | | 1.66 | | 0.2073 | |
|  |  |  |  | (-0.89 - 3.47) | |  | | (-0.51 - 3.84) | |  | |
| Menthol Vuse Solo 36 mg | 17.15 | | | 0.99 | | 0.4605 | | 1.36 | | 0.3054 | |
|  |  |  |  | (-1.22 - 3.20) | |  | | (-0.83 - 3.56) | |  | |
| Usual Brand Cigarette | 16.16 | | |  | |  | |  | |  | |
| Nicotine Gum | 15.79 | | |  | |  | |  | |  | |

Supplemental Table 5. Baseline-adjusted plasma nicotine parameters from current menthol and previous non-menthol studies

|  | **Vuse Solo**  **14 mg** | | **Vuse Solo**  **29 mg** | | **Vuse Solo**  **36 mg** | | **Usual Brand**  **Cigarette** | | **Nicotine Gum** | |
| --- | --- | --- | --- | --- | --- | --- | --- | --- | --- | --- |
| **Parameter**^1^ | **Menthol** | **Non-Menthol** | **Menthol** | **Non-Menthol** | **Menthol** | **Non-Menthol** | **Menthol** | **Non-Menthol** | **Menthol** | **Non-Menthol** |
| **C_max_ (ng/mL)** | 2.45^*,†^ | 3.01 | 3.40^*^ | 4.67 | 3.94^*^ | 5.36 | 18.04 | 17.98 | 4.80 | 5.26 |
| **AUC_nic0-15_ (ng*min/mL)** | 17.14^*,†^ | 22.30 | 26.26^*,†^ | 42.64 | 33.14^*,†^ | 37.30 | 176.30 | 180.72 | 8.44 | 5.89 |
| **AUC_nic0-360_ (ng*min/mL)** | 412.34^*,†^ | 482.39 | 545.14^*,†^ | 642.70 | 516.15^*,†^ | 658.97 | 1556.44 | 1670.32 | 844.01 | 884.22 |
| **T_max_ (min)** | 19.89^*^ | 27.35 | 15.10^*^ | 21.83 | 10.13^*,†^ | 24.17 | 7.43 | 8.13 | 45.04 | 50.88 |
| ^1^ C_max_ and AUC parameters presented as geometric LS means; T_max_ presented as median.  ^*^ Statistically significantly different than Usual Brand Cigarette, p <0.0005  ^†^ Statistically significantly different than Nicotine Gum, p<0.0005 | | | | | | | | | | |
